# Annotations

**Published:** 1901-09-14

**Authors:** 


### ANNOTATIONS.

### Operations by House-Surgeons.

A REGULATION recently promulgated at one of the largest hospitals in America shows how difficult it is to lay down general rules, and how easy to jump to absurd extremes when attempts are made to impose limitations upon the ever-varying duties of medical men. At Cook County Hospital, which we may fairly call the great hospital of Chicago, a stringent order has been issued prohibiting the performance of what are spoken of as major operations by the internes, either with or without the presence of the attending surgeon, setting forth that "in any case of emergency the house-physician or house-surgeon shall secure the immediate attendance of the attending physician or surgeon," and adding, as a definition of "major operation," that "this rule covers all operations where it becomes necessary to place patients under anæsthetics." We do not know the exact conditions under which the internes at the Cook County Hospital are appointed, but if they are selected as are the house-surgeons and resident medical officers of the great hospitals in London, *i.e.*, from the most prominent and gifted of those who have studied at their schools, we have no hesitation that the rule mentioned is an absurd one. No doubt in London the rule as to the performance of operations varies a good deal, and at some hospitals the surgeons are more liberal in their dealings with their house-surgeons than at others. But at no hospital could we conceive it possible for such a rule as the one to which we have referred to be put in force without inflicting great cruelty and injury upon the patients. Imagine a hospital where 67,000 minor casualties are treated in the surgery in the course of a year, besides those which are severe enough to require admission to the wards, having to send cabs trotting across London at all hours of the day and night, to fetch members of the staff every time an anæsthetic happened to be wanted. What is essential is that every large hospital should have a thoroughly capable resident staff. What the members of this staff are to be allowed to do must be arranged between them and their senior officers. It must often vary with the individuals; it may vary, as no doubt it does, even with the time of year, but any broad rule such as the one to which we now draw attention, which, while appealing to the public as being for the advantage of the patients would really act greatly to their detriment by introducing delay and excuses for delay at every turn, is greatly to be deprecated in any well-managed institution.

### The Medical Guild and the Midwives.

THE question of the midwives still continues to be prominently brought before the profession by the advocates of the various schemes which have been proposed for its solution. It cannot be said, however, that much advance is being made towards harmony of action or unanimity of opinion. Indeed for a time the angry passions which this subject seems always to arouse have been diverted into new channels, interest just now being especially centred in the question of priority of authorship in regard to one of the proposals now in market. The Medical Guild has propounded a scheme without any reference apparently to the fact that three years or so ago Dr. McCook Weir drew up a Bill something like it. Hence trouble and disputation. Whether the medical profession, which Dr. Weir not altogether untruthfully describes as a "helpless rabble," will really have any material say in the decision when Parliament ultimately takes the matter in hand may be open to question, but at the present moment the suggestion which seems to receive the largest share of professional support, so far as talk is concerned, is this said proposal of the Medical Guild, the aim of which is that no one should be legally recognised as fitted to conduct cases of midwifery except under the direct and personal supervision of those who are registered under the Medical Acts. This is a very definite proposition, and looking at the matter purely from the professional side, it has our every sympathy. It would sweep into the medical net the whole art and mystery of midwifery, and however the matter of fees might be arranged, it could hardly fail to bring in grist to the mill. When, however, things come before Parliament it is astonishing how different they look. The liberty of the subject, the "rights" of those at present engaged in midwifery work, the rivalry between the two professions—the doctors and the midwives to wit!—and the possible tyranny of "medical trades-unionism," besides the obvious financial difficulties, will all be trotted out, and when the Bill has gone through committee, it is quite probable that even its own parents will hardly know it again. All of which is a pity, for it would be really nice to have the whole matter in our own hands!

### Pharmacomania.

THE habit of drug-taking is easily acquired but with difficulty suppressed, for whatever may be the initial trouble from which relief is sought, the action of the drug is immediate and therefore invites repetition. Unfortunately, the weak-nerved people, who seem to be terrified at the idea of a sleepless night and collapse altogether if attacked with a toothache, are those who soonest fly to narcotics and other soothing remedies and have not the strength of mind to struggle against and finally conquer the habit when once acquired. Again, there is a class of people, fortunately a small one, who have a positive mania for trying every new drug which appears, with the result that in a short time their digestive organs are ruined and their nervous tone lowered, with the result that they have resource to still more drugs, and thus the vicious circle is complete. The effects of habitually taking large quantities of such drugs as opium, chloral and cocaine are not however limited to the physical degradation of the individual, but also lead to mental incompetency and loss of the moral sense. Even after voluntarily submitting themselves to treatment and being genuinely anxious to make an effort, the habit is often too strong, and the most ingenious devices are practised to deceive the doctor and nurse. It is obvious that it is impossible to control such patients unless they submit to absolute confinement, as is now possible in the case of habitual drunkards. Habitual drunkards are far more numerous, and thus a greater nuisance to society, but it is doubtful whether an opium-besotted head of a family, with power to fritter away his property, is not an equal misfortune to his immediate relations. The Reformatory for Inebriates at Farmfield established by the London County Council in 1899 has been a decided success, and in course of time we may hope that society may be freed from the spectacle of hopeless drunkards altogether. In the meantime it is necessary to draw attention to the fact that nothing is being done for the victims of opium, nepenthe, chloral and cocaine who are equally deserving of our commiseration.

